# Research Progress in Understanding the Relationship Between Heme Oxygenase-1 and Intracerebral Hemorrhage

**DOI:** 10.3389/fneur.2018.00682

**Published:** 2018-08-20

**Authors:** Qian-Qian Li, Lan-Jun Li, Xin-Yu Wang, Yu-Ying Sun, Jun Wu

**Affiliations:** Department of Neurology, The First Affiliated Hospital of Zhengzhou University, Zhengzhou University, Zhengzhou, China

**Keywords:** heme oxygenase-1, intracerebral hemorrhage, heat shock protein 32, heme, neurological impairment, microglia

## Abstract

Intracerebral hemorrhage (ICH) is a fatal acute cerebrovascular disease, with a high morbidity and mortality. Following ICH, erythrocytes release heme and several of its metabolites, thereby contributing to brain edema and secondary brain damage. Heme oxygenase is the initial and rate-limiting enzyme of heme catabolism, and the expression of heme oxygenase-1 (HO-1) is rapidly induced following acute brain injury. As HO-1 exerts it effects via various metabolites, its role during ICH remains complex. Therefore, in-depth studies regarding the role of HO-1 in secondary brain damage following ICH may provide a theoretical basis for neuroprotective function after ICH. The present review aims to summarize recent key studies regarding the effects of HO-1 following ICH, as well as its influence on ICH prognosis.

## Introduction

Intracerebral hemorrhage (ICH) is in general referred to as non-traumatic spontaneous hemorrhage within the brain parenchyma; it is the second most common cause of stroke, representing approximately 10–15% of all stroke cases (1–5). Relative to ischemic stroke, ICH is associated with increased morbidity and mortality, severely impairing physical and mental health and negatively influencing patient quality of life (6–9). Secondary neurological impairment after ICH is an important cause of the aforementioned phenomena. Degradation of hemin—the oxidized form of heme released by erythrocytes following ICH—can lead to cell and tissue trauma via direct cytotoxic effects, the release of redox-active iron, and the depletion of cellular NADPH and glutathione stores (10). Previous studies have further reported that hemin can enter brain cells (10–13), contributing to brain edema and secondary neurological damage by inducing inflammatory reactions and damaging the blood brain barrier (BBB) (13–15). Because hemin cannot circulate within the central nervous system (CNS), accelerating hemin catabolism may promote hematoma clearance following ICH (16, 17).

Heme oxygenase is the initial and rate-limiting enzyme of heme catabolism. Three isoenzymes of heme oxygenase exist: HO-1, HO-2, and HO-3 (18) (Table 1). HO-1 is an inducible isoform, which has been extensively studied primarily for its ability to respond to oxidative stress, hemorrhage, and trauma (19–21). Previous studies have demonstrated that HO-1 is rapidly induced following ICH, and that HO-1 activators can alleviate BBB dysfunction and neurological impairment after ICH (22, 23). HO-2, the constitutive isoform, is diffusely distributed in the brain and testes (25). Previous reports have indicated that HO-2 can be induced by glucocorticoids as well as oxygen (26–29, 32, 33). The physiological role of HO-3 is not fully understood, but it most likely stems from the effects of HO-2 (30, 31). Researchers have concentrated their attention on the effects of HO-1 due to its potential as a target for intervention in ICH. In this review, we summarize recent studies focused on the relationship between HO-1 and ICH, including the role of HO-1 in secondary brain damage following ICH. We further discuss the vital role of HO-1 in ICH research, emphasizing the importance of identifying novel therapeutic targets for ICH.

**Table 1:** The isoforms of heme oxygenase.

**Isoforms**	**Chromosome**	**Gene**	**Molecular weight (Da)**	**Distribution**	**Activators**	**References**
HO-1	22q12	*HMOX1*	32,000	Primary in vascular-like structures, but at low levels in the CNS	Hemoglobin Heme Metals Cytokines Oxidative stress Hypoxia Glutathione Ultraviolet irradiation Lipopolysaccharide CoPP Endotoxin Nitric oxide	(19–24)
HO-2	16p13.3	*HMOX2*	36,000	Diffusely distributed in the brain and testis	Glucocorticoids O_2_	(25–29)
HO-3	/	/	/	Most likely stems from HO-2	/	(30, 31)

## Biological characteristics of HO-1

HO-1 is encoded by the *HMOX1* gene, which is located on chromosome 22 and contains four introns and five exons (34). HO-1, also known as heat shock protein 32 (HS32), is an inducible heme oxygenase with 288 amino acids and a molecular weight of 30,000–33,000 Da (24, 35, 36) (Table 1). The HO-1 promoter contains binding sites for various transcription factors such as heat-shock factor, nuclear factor kappa B (NF-κB), activator protein 1 (AP-1), and metal regulatory elements (MREs) (37–40). Originally identified as a liver microsomal protein (41, 42), HO-1 is widely present in systemic tissues, especially in microsomes of the mononuclear macrophage system. The highest HO-1 contents have been observed in the liver, bone marrow, and spleen. Under physiological conditions, HO-1 is expressed primarily in vascular-like structures, but at low levels in the CNS. Expression of *HMOX1* is induced in response to a variety of endogenous and exogenous oxidative and inflammatory signals, including hemoglobin, heme, metals, oxidative stress, hypoxia, ultraviolet irradiation, heat, nitric oxide, endotoxin, lipopolysaccharide, cytokines, cobalt protoporphyrin-IX, and glutathione (32, 43) (Table 1). Nuclear transcription factor-erythrocyte 2 related factor (Nrf2) regulates transcriptional activation of *HMOX1* (44, 45).

HO-1 is a cytoprotective molecule that is crucial in maintaining cell homeostasis. HO-1 exerts strong anti-oxidant and anti-inflammatory effects by promoting heme catabolism to produce CO and bilirubin. However, it can also produce reductive ferrous iron, and therefore may be cytotoxic (46). Previous studies have revealed that HO-1 exerts strong protective effects in preclinical models of several diseases, including cardiovascular and cerebrovascular disease, diabetes, sepsis, trauma, vascular proliferative diseases, acute lung injury, liver injury, gut ischemia/reperfusion injury, and tumors (47, 48). The following section mainly focuses on the role of HO-1 in ICH.

## HO-1 and ICH

### Changes in HO-1 expression after ICH

#### Preclinical studies

**Figure 1:**
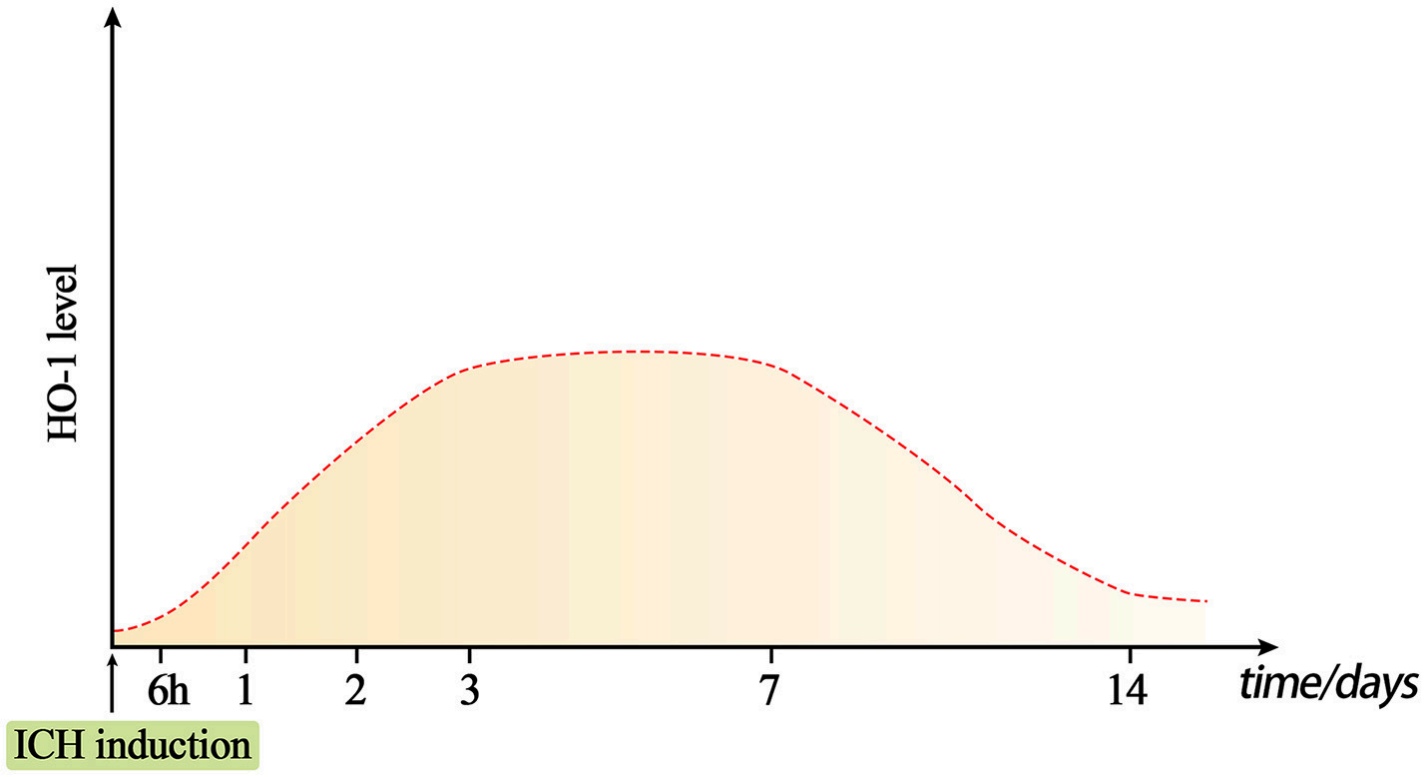
Dynamic changes in HO-1 levels after intracerebral hemorrhage (ICH). Under normal conditions, HO-1 content in the brain is very low. The expression of HO-1 can be rapidly induced approximately 6 h after ICH, and reaches a peak at days 3–7. The level of HO-1 may gradually decrease on day 7, but it will remain present for at least 14 days.

Numerous studies have verified that HO-1 expression is rapidly induced after acute brain injury in mouse, rat, and rabbit models of traumatic brain injury (TBI), subarachnoid hemorrhage (SAH), and ICH induced by collagenase or autologous blood injection (49–53) (Table 2). Okubo et al. (54) investigated cerebral hematoma development, brain edema formation, BBB disruption, and HO-1 expression after TBI. In their study, elevated HO-1 levels and iron deposition were observed in the ipsilateral hemisphere at 24 h after craniocerebral injury. In addition, the expression of HO-1 in the rat brain began to increase as early as 6 h after an open-skull, weight-drop-induced TBI, increasing continuously during the investigation (55) (Figure 1). These findings suggest that hematoma development upregulates the expression of HO-1 following TBI, which also promotes heme decomposition and iron deposition. Increased expression of HO-1 has also been observed in mouse models of SAH (63). Mouse models of acute ICH developed via the injection of autologous blood into the striatum exhibited increases in HO-1 levels within the first 24 h, exhibiting a 10-fold increase relative to baseline by day 5 and returning to baseline levels on day 8 (57). Similar results were confirmed in collagenase-induced mouse models of ICH (Figure 1): On the first day after ICH, the expression of HO-1 protein significantly increased in the ipsilateral striatum, peaking on day 3 and decreasing by day 7. On days 14 and 28 after ICH, the expression of HO-1 did not significantly differ from that observed in the control group (14).

**Table 2:** Summary of HO-1 in preclinical and clinical studies of intracerebral hemorrhage.

**Study types**	**Models**	**Species**	**Expression cell**	**HO-1 expression start time**	**HO-1 level peak time**	**Effect**	**References**
Preclinical	TBI	Male SD Rats	Microglia	24 h	/	/	(54)
	TBI	Lewis rats	Microglia/Macrophage (CD163^+^/CD68^+^)	6 h	Day 3	Anti-inflammatory	(55)
	Autologous blood injection	Male and female SD Rats	Microglia	24 h	Day 3–day 7	Early protection and late damage	(56)
	Collagenase injection	Swiss-Webster Mice	/	24 h	Day 5	Not clarified	(57)
	Autologous blood injection	Male SD Rats	Microglia (CD11b^+^)	6 h	/	Early protection	(58)
	Collagenase injection	C57BL/6 mice	Microglia and neurons	24 h	Day 3–day 7	Early protection and late damage	(14)
	Collagenase injection	HO-1-/-Mice and WT-mice (male)	Microglia and endothelial cells	5 h	/	Exacerbates early brain injury	(59)
	Collagenase injection	GFAP.HMOX1 Mice(Male and female)	Astrocyte	7 h	/	Protection	(60)
	Autologous blood injection	Male SD rats	/	8 h	Day 5	A sensitive marker of ICH brain injury.	(61)
	Autologous blood injection	Male New Zealand White Rabbits	Microglia and astrocyte	24 h	/	Early protection, and late damage	(62)
	Subarachnoid Hemorrhage (SAH)	Male C57BL/6 Mice	Microglia	/	/	Protection	(63)
	**Disease**	**Patients age**	**Distribution**	**Time**		**Effects**	**Reference**
Clinical	TBI	Infants and children	CSF	One time point < 24 h and one time point >24 h		/	(64)
	SAH patients with vasospasm	Age 32–72 years	CSF	/		/	(65)
	Fisher grade III SAH	Mean age 59 ± 14 years	CSF	Day 7		HO-1 with higher values predicting unfavorable outcome	(66)
	ICH	Adults (40–70 years old)	CSF	Within 7 days		/	(67)

*ICH, intracerebral hemorrhage; TBI, traumatic brain injury; SAH, subarachnoid hemorrhage; CSF, cerebrospinal fluid*.

Similar results have been obtained using rat models. Wang and colleagues (56) established a rat model of ICH via the injection of autologous blood. In this model, the number of HO-1-positive cells around the hematoma increased as early as 1 day after ICH. Furthermore, HO-1 mRNA could be detected around the hematoma beginning on day 3, reaching a plateau at days 3 and 7 after ICH and lasting until at least 14 days after ICH. Researchers also observed that HO-1 levels begin to increase significantly at 8 h following autologous blood injection in rat models of ICH, and that such increases in HO-1 are closely related to brain edema and neurological impairment, which may reflect the severity of brain injury to some extent (61). Similar results have also been observed using rabbit models of ICH induced via autologous blood injection (62).

#### Clinical studies

In one clinical study involving 48 infants and children with TBI and seven healthy controls, researchers reported that the patient group exhibited increases in cerebrospinal fluid (CSF) levels of HO-1 following TBI, and that such increases were more prominent in infants than in older children (64). Additional studies have reported that CSF levels of HO-1, bilirubin, and peroxidized lipids are significantly higher in patients with SAH who experience vasospasm than in those without vasospasm (65). Although Li et al. (67) were the first to report that serum levels of HO-1 are higher in patients with ICH than in those without, it remains to be clarified whether HO-1 exerts protective or harmful effects in patients with ICH. Additional researchers have investigated the prognostic value of CSF biomarkers related to heme metabolism, including HO-1, oxyhemoglobin, ferritin, and bilirubin in 41 patients with Fisher Grade III aneurysmal SAH. Their study indicated that CSF levels of HO-1 at day 7 post-SAH can be a potent prognostic indicator in this patient population (66).

The results of the aforementioned studies suggest that HO-1 expression is rapidly induced following ICH, likely via one or more of the following mechanisms: (1) Heme released by damaged erythrocytes after ICH directly induces HO-1 expression; (2) HO-1 expression is induced by ischemia and hypoxia around the hematoma after ICH; (3) oxidative stress after ICH leads to upregulation of HO-1 expression via the Nrf2-ARE signaling pathway. As such, HO-1 may represent a useful biomarker of damage due to heme-mediated oxidative stress following ICH (61). Furthermore, the *HMOX1* promoter region contains several sites, and various stimuli may act on this region in conjunction with heme to induce *HMOX1* gene expression (37, 38, 39, 40). However, the exact molecular mechanisms underlying gene-induced increases in the expression of HO-1 following ICH remain to be elucidated. Further in-depth studies are required to determine how changes in HO-1 expression following ICH can be used to improve the prognosis of patients with ICH.

### The effect of HO-1 expression at different stages after ICH

Although previous studies have demonstrated that HO-1 is rapidly induced after ICH, the role of HO-1 in the early and late stages of ICH remains controversial. Wang et al. (56) investigated collagenase-induced ICH in a mouse model, reporting that HO-1 exerts both anti-oxidant and pro-oxidant properties in ICH. Their results indicated that increases in HO-1 expression on days 1–7 after ICH play a protective role. Moreover, RT-PCR results revealed a positive correlation between copper–zinc superoxide dismutase (Cu/Zn-SOD) and HO-1. Cu/Zn-SOD is a key antioxidant enzyme involved in the detoxification of SOD in the metabolism of normal cells, playing a role in antioxidative stress and exerting cytoprotective effects. Similarly, in other diseases of the CNS (e.g., Parkinson's disease, Alzheimer's disease, etc.), HO-1 acts in conjunction with Cu/Zn-SOD to exert protective effects against oxidative stress (68–70). Conversely, in the late stage of ICH (day 7), overexpression of HO-1 aggravates neurological deficits. RT-PCR have further revealed that HO-1 levels are positively correlated with those of malondialdehyde (MDA), but not with Cu-/Zn-SOD content. MDA is a product of lipid peroxidation, which can represent the production of peroxy radicals. MDA levels can also reflect the degree of oxidation *in vivo*. Thus, at different times after ICH, positive correlations between HO-1 and Cu-/Zn-SOD dismutase or MDA, respectively, may exert protective and detrimental effects on oxidative stress. Therefore, appropriately up-regulating HO-1 expression may play a neuroprotective role in the early stage of ICH, while down-regulation of ICH expression in later stages may attenuate neurological impairments. Another study demonstrated that HO-1 levels begin to increase 6 h after ICH, exerting neuroprotective effects from 12 h to 7 days. These results suggest that the neuroprotective effects of HO-1 are triggered in the early stages of ICH, and that such effects may be associated with HO-1 induced regulation of Nrf2 and NF-κB entry into the nucleus (58).

However, in one recent study, Zhang et al. (14) investigated the effects of an HO-1 inducer and inhibitor in a collagenase-induced mouse model of ICH. They observed that HO-1 exacerbated brain injury in the early stages of ICH (1–3 days) but promoted hematoma absorption and recovery of neurologic function in the later stages (7–28 days). The authors further reported that HO-1 aggravated brain damage via multiple mechanisms, including microglial activation, BBB damage, inflammatory reactions, neuronal cell death, oxidative damage, white matter injury, and iron accumulation. However, in the late stages of ICH, HO-1 played a neuroprotective role by increasing hematoma absorption and angiogenesis. Additional studies involving HO-1 knockout mice have indicated that HO-1 may exacerbate early neurological impairment after ICH (59).

Results regarding the effects of HO-1 expression at different stages of ICH remain controversial, likely due to factors such as the model selected (collagenase vs. autologous blood injection) and the method of treatment (e.g., anticoagulant heparin, HO-1 inducers and inhibitors, etc.) Future multi-center animal studies should utilize standardized experimental methods to obtain more reliable preclinical results.

At present, there is no effective treatment for ICH. While recombinant factor VIIa (rFVIIa) therapy (71–74), minimally invasive intracranial hematoma removal (75), immunosuppressant therapy (e.g., fingolimod) (74, 76–79), nanomedicine (80, 81), stem cell transplantation (82–86), and treatment with anti-neuroinflammatory, anti-oxidative, and neuroprotective agents (87–89) may produce some therapeutic effects, such treatments have failed to achieve breakthrough results. Some researchers have speculated that there may be a “time window” for effective treatment for ICH, similar to that observed for ischemic stroke. However, such a time window has yet to be defined. Initial studies have revealed that HO-1 expression is rapidly induced following ICH, and that dynamic changes in HO-1 levels can be observed at different stages of ICH, along with differences in the protective and detrimental effects of HO-1. Further studies are required to elucidate the mechanisms underlying the expression of HO-1 after ICH, the time-point at which HO-1-targeted treatments should be administered, and potential biomarkers for predicting patient prognosis. Targeting HO-1 expression after ICH may help to alleviate secondary brain damage and improve prognosis, although further studies regarding the effects of HO-1 during the early and late stages of ICH are required.

### Effect of HO-1 expression on ICH

**Figure 2:**
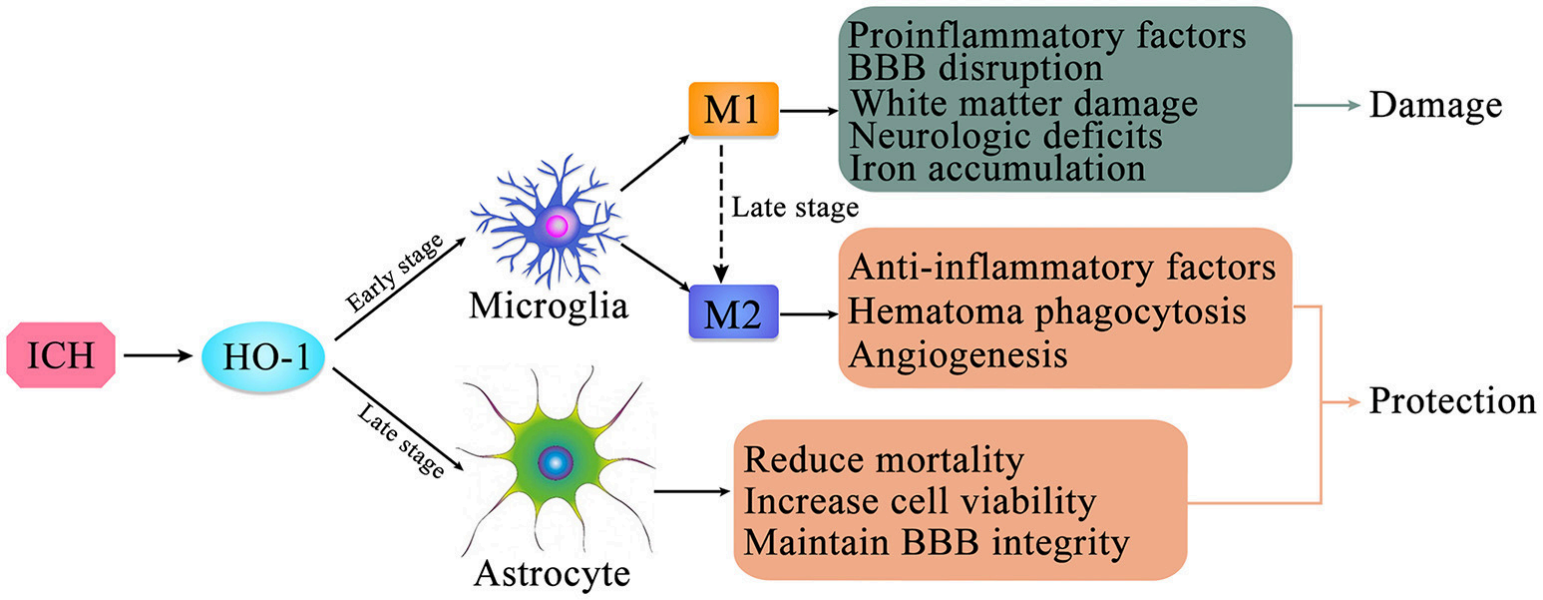
The expression of HO-1 in microglia and astrocytes after intracerebral hemorrhage (ICH). HO-1 is expressed in both microglia and astrocytes after ICH. HO-1 is mainly expressed in microglia in the early stage of ICH; it is primarily expressed in astrocytes in the late stage of ICH. Microglia can be polarized to two phenotypes, M1 and M2, which are in dynamic flux during ICH. After ICH, M1 microglia can produce pro-inflammatory factors, leading to destruction of the blood-brain barrier, brain edema, white matter damage, iron accumulation, and neurological deficits; M2 microglia can produce anti-inflammatory factors and promote hematoma clearance through phagocytosis and promotion of angiogenesis. In the late stage of ICH, high level HO-1 expression in astrocytes may contribute to increased cell viability, maintenance of blood-brain barrier integrity, and reduction of mortality.

Previous studies have shown that, after ICH, HO-1 is expressed in both microglia (55, 90–92) and astrocytes (45, 93, 60) (Figure 2), which exert distinctly different effects on ICH (14, 44, 60). Recent research has focused primarily on the relationship between HO-1 expression and microglia, while studies regarding the relationship between HO-1 expression and astrocytes remain relatively rare and controversial.

Microglia, which are often referred to as phagocytes in the brain, are the primary immune effector cells of the CNS. Microglia can exert both neurotoxic and restorative effects, depending on the type of stimulation, intensity and duration of the stimulus, microenvironment, interactions with other cells, and patient age (87). Zhang et al. (14) revealed that HO-1 is abundantly expressed in microglia in the early stages after ICH, increasing microglial activation and aggravating neurological impairments. The authors performed immunofluorescence experiments to detect changes in HO-1 localization in a rat model of TBI, observing that HO-1 levels began to increase at 6 h after TBI, reaching a peak at 96 h. Immunofluorescence double-staining revealed that most HO-1 cells co-expressed CD68 and CD163, but that HO-1 was not expressed in astrocytes [based on glial fibrillary acidic protein (GFAP) expression]. Therefore, HO-1 expression may define a subtype of activated microglia/macrophage with a specific role in the anti-inflammation processes following TBI (55). In addition, in the early stage of ICH, HO-1 is mainly upregulated in microglia and endothelial cells. Compared with wild-type mice, HO-1 knockout exerts a significant protective effect on early brain function following ICH. These results suggest that this protective effect is related to a reduction in microglia activation, leukocyte infiltration, and the production of reactive oxygen species (ROS) in the early and critical stage after induction of ICH (59). Moreover, the neurological function of wild-type mice improved over time, while the neurological function of HO-1 knockout mice remained constant, suggesting that HO-1 exerted a protective effect in the wild-type group during the ICH recovery phase. Conversely, Schallner et al. (47) reported that microglia regulate blood clearance in SAH via HO-1. Furthermore, the authors reported that microglial HO-1 is necessary for attenuating neuronal cell death, vasospasm, impairments in cognitive function, and for clearance of the cerebral hematoma.

Collectively, these findings indicate that microglial activation following ICH is closely associated with HO-1. Although HO-1 is abundantly expressed in microglia, its role following ICH remains controversial (Figure 2). Previous studies have confirmed that microglial activation occurs following ICH, developing the classic M1-like (pro-inflammatory) or alternative M2-like (anti-inflammatory) phenotype (94, 95), which may fluctuate during the course of ICH. Following ICH, M1 microglia produce a large number of pro-inflammatory factors, leading to destruction of the BBB, brain edema, white matter damage, and neurological deficits. In contrast, M2 microglia produce anti-inflammatory factors and promote hematoma clearance through phagocytosis and angiogenesis (96). However, the detailed mechanisms underlying microglial activation and polarization after ICH remain to be clarified. Further studies regarding the role of microglial in secondary brain injury after ICH may provide insight into the neuroprotective effects of microglial cells. Moreover, inhibiting microglial activation may reduce neurotoxicity and improve the clinical prognosis of patients with ICH.

To date, no studies have investigated the effects of HO-1 expression on M1/M2 polarization and ICH prognosis. Thus, conclusions cannot be drawn based on existing studies. Future studies should focus on the effect of HO-1 expression on microglial polarization following ICH, and whether HO-1 expression differs between M1/M2 microglia.

Several studies have investigated the relationship between HO-1 and astrocytes. Studies involving GFAP·HMOX1 mice [driven by the glial fibrillary acidic protein (GFAP) promoter] have calculated the volume of ICH, mortality, and neurological impairment to evaluate the role of HO-1 after ICH. Such studies have demonstrated that overexpression of HO-1 in astrocytes exerts a strong neuroprotective effect after ICH and improves ICH prognosis (44, 60, 97). Furthermore, Chen-Roetling et al. (44) suggested that selective HO-1 expression in astrocytes reduces mortality, BBB destruction, hematoma-peripheral cell damage, and neurological deficits after ICH (Figure 2).

Kazuhiro et al. used immunohistochemistry to investigate the expression and localization of HO-1 in a rat model of ICH developed via autologous blood injection. Their results revealed that microglia and astrocytes co-induced the expression of HO-1 after ICH. HO-1 was mainly expressed in microglia during the acute phase (1–7 days), and in astrocytes during the subacute and chronic phases (7 days later) (93). Additional studies involving adult rabbits have verified that HO-1 is mainly expressed in microglia following acute ICH and in astrocytes during the late stages of ICH, while expression in neurons and oligodendrocytes is less common (62). Interestingly, astrocytes are also involved in microglia M1 to M2 phenotype conversion after ICH (98, 99), with a complicated internal mechanism (Figure 2).

Taken together, these findings demonstrate that the effects of HO-1 induction on nerve function differ substantially according to cell type. Further in-depth studies are required, regarding the effects of astrocytes on the function of microglia polarization, phagocytosis, angiogenesis, and other functions, to determine whether mechanisms other than those involving microglia and astrocytes play a role in HO-1 expression during ICH. These will greatly improve our understanding of ICH pathology and may provide novel strategies for follow-up treatment of ICH.

### The role of catalytic decomposition products of HO-1 after ICH

**Figure 3:**
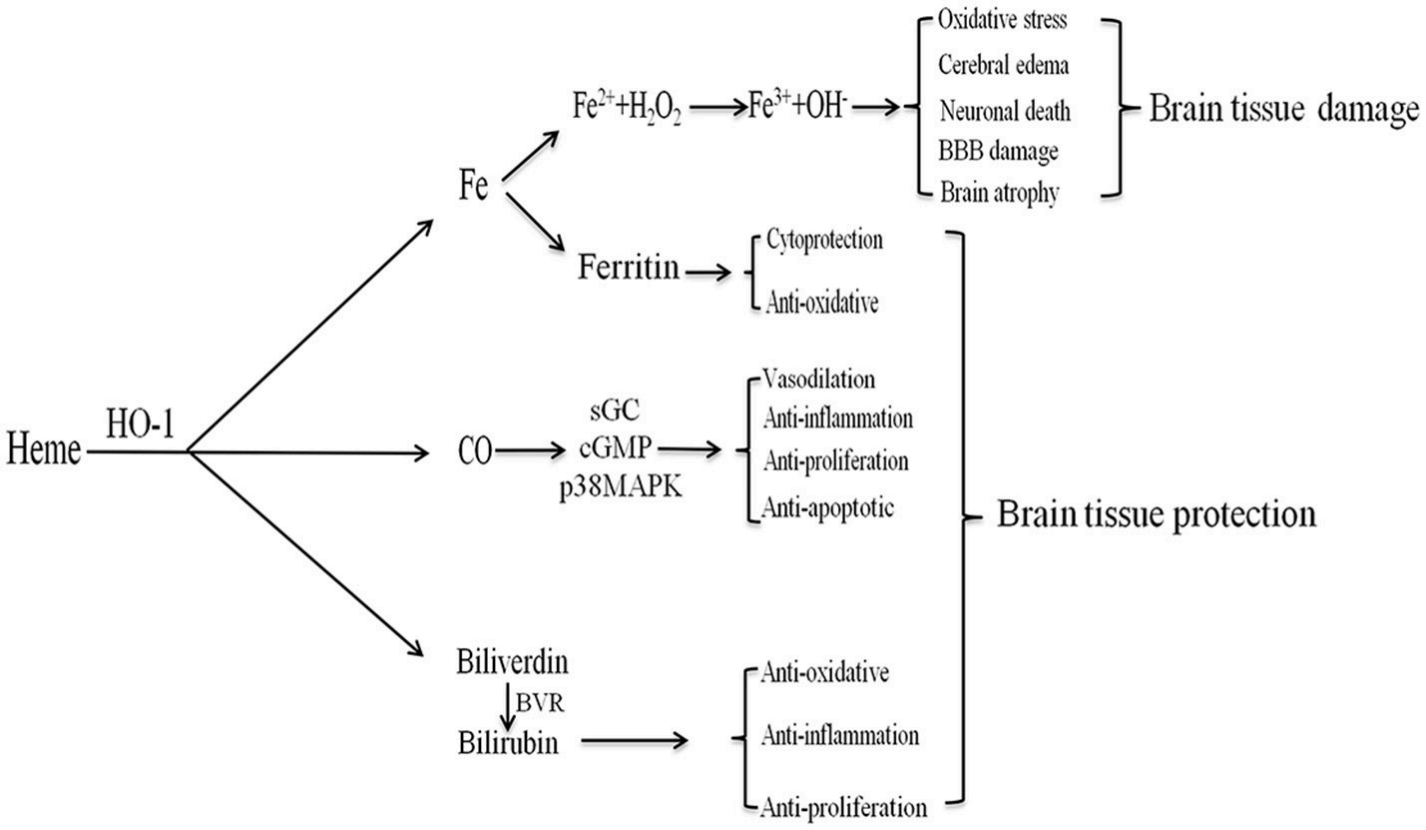
The products of heme metabolism catalyzed by HO-1 (heme oxygenase (HO-1) metabolizes heme). After intracerebral hemorrhage (ICH), HO-1 can catalyze heme to produce biliverdin, carbon monoxide (CO), and free iron. Iron can produce ferritin to exert cytoprotection and anti-oxidative effects; it can also be oxidized to Fe^3+^, which contributes to brain edema, oxidative stress, neuronal death, blood-brain barrier damage, and brain atrophy. CO can play anti-apoptotic, anti-inflammatory, anti-proliferative, and vasodilating effects through the sGC/cGMP and p38MAPK pathways. Biliverdin is rapidly converted to bilirubin under the BVR, which has anti-inflammatory, anti-oxidative and anti-proliferative effects.

The development of brain edema following ICH is a complex pathophysiological process involving several mechanisms, including toxicity associated with heme degradation-related products catalyzed by HO-l. HO-1 can catalyze the production of biliverdin, CO, and free iron (Figure 3).

#### Biliverdin

HO-1 catalyzes free heme and produces equimolar biliverdin, which is rapidly converted to bilirubin via a catalytic reaction involving biliverdin reductase (31). Bilirubin, the most abundant endogenous antioxidant in human serum, is significantly elevated in patients with ICH (100). Furthermore, bilirubin can scavenge free radicals, inhibit the modification of low-density lipoprotein oxidation by free radicals, resist lipid peroxidation, and exert anti-inflammatory, anti-proliferation, and cytoprotective effects (32, 28, 101). The serum bilirubin level reflects the intensity of oxidative stress and HO-1 expression in response to oxidative stress in various diseases (100). However, tissue damage occurs when the heme content is high and when hyperheme is formed, exerting a detrimental effect on prognosis in patients with ICH. As the role of bilirubin after ICH is 2-fold, it is therefore important to moderately regulate bilirubin expression while avoiding potential hazards when investigating its impact on brain function following ICH.

#### Co

CO is an important gas conductor and vasodilator with anti-apoptotic, anti-inflammatory, and anti-proliferative properties (43, 102, 103). Mice pretreated with CO exhibit reduced production of the serum inflammatory cytokines tumor necrosis factor α (TNF-α), interleukin 1β (IL-1β), and IL-6, along with increased production of the anti-inflammatory cytokine IL-10. In addition, these mice exhibited reduced organ damage and prolonged survival time (104). CO can play a protective role by regulating multiple signaling pathways, including the soluble guanylate cyclase (sGC) and mitogen-activated protein kinase (MAPK) pathways (105–107). The p38MAPK pathway in particular is closely associated with anti-inflammatory, anti-apoptotic, and anti-proliferative effects (105, 104, 108–110). In addition, CO lowers blood pressure by regulating the release of neurotransmitters. HO-1/CO also plays an important role in the central regulation of pulse pressure in the moving axis (32). Schallner et al. (47) demonstrated that the HO-1/CO axis increases the phagocytic activity of microglia and promotes erythrocyte clearance following ICH. When HO-1 is limited in microglia, phagocytosis is impaired, resulting in increased neuronal damage and cognitive impairment. These effects can be attenuated via the administration of exogenous CO. The HO-1/CO axis may also play an immunomodulatory role in regulating the function of antigen-presenting cells, dendritic cells, and regulatory T cells (43).

Taken together, these studies indicate that the HO-1/CO axis plays an important role in the subsequent pathophysiological changes associated with ICH. Regulation of the HO-1/CO axis to alleviate damage to nerve functions after ICH may represent a novel target for achieving neuroprotection following ICH.

#### Iron

Iron, which is catalyzed by HO-1, is another product of heme degradation. Iron is an essential cofactor for various cytochromes and redox-dependent proteins. However, numerous studies have indicated that iron overload can cause brain damage and irreversible neurological deficits through various pathways after ICH, including lipid peroxidation and the formation of free radicals (111–114). Iron deposition can occur within a short period of time after ICH, lasting at least 14 days in many cases (115, 116). Additional studies have demonstrated that intracerebral iron overload can cause cerebral edema, oxidative stress, BBB damage, neuronal death, brain atrophy, and combined ischemia after ICH (117). In addition, iron-induced oxidation can lead to DNA damage in the brain after ICH, and may be a direct cause of post-ICH edema. HO-derived iron can exert both anti-oxidative and cytoprotective effects. Indeed, HO-1-induced increases in ROS production and lipid oxidation are due in part to iron accumulation during the early stages of ICH. One MRI study revealed that iron content in the brain and hematoma volume influence the extent of the surrounding edema after ICH (118). Therefore, inhibiting iron accumulation may attenuate delayed injury following ICH, representing a promising target for ICH therapy (54, 119).

Deferoxamine (DFO) is an iron chelator and an inhibitor of microglial activation. Previous studies have confirmed that DFO can reduce secondary impairments in neurological function due to ICH (e.g., brain edema, neuronal death, brain atrophy, and neurological deficits) in young rats (101, 120, 121), aged rats (122, 123), pigs (124), and patients (74, 125, 126). Studies have also revealed that both iron overload and aquaporin 4 (AQP4) play a key role in the development of brain edema following ICH. Moreover, such studies have indicated that AQP4 expression is influenced by iron concentration. Notably, DFO treatment significantly inhibits AQP4 upregulation and reduces cerebral edema in rat models of ICH (119, 120). Minocycline, another iron-chelating agent, can exert neuroprotective effects by attenuating microglial activation and matrix metalloproteinases in the CNS in models of stroke, TBI, and neurodegenerative disease (127, 128). Previous researchers have also reported that minocycline can reduce iron overload and iron-induced neurological deficits after ICH in male rats (128, 129). Recent studies have further demonstrated that minocycline attenuates secondary brain injury and iron overload after ICH in aged female rats (6). Importantly, early clinical trials reported that a 400 mg dose of minocycline was safe and achieved neuroprotective serum concentrations in patients with ICH (130). Additional studies have indicated that valproate (VPA) may also play a neuroprotective role by down-regulating the expression of HO-1, thereby reducing iron release (12).

Taken together, these findings indicate that iron plays a vital yet complex role in functional neurological impairments following ICH. Iron-chelating agents can attenuate cerebral edema, neurological deficits, and brain atrophy following ICH. Indeed, the prevention of iron-mediated toxicity represents a promising therapeutic strategy for ICH (15, 131).

### Anti-inflammatory and antioxidant effects of HO-1 after ich

The inflammatory response after ICH plays an important role in brain edema and is positively correlated with disease severity (132). NF-κB is an important transcription factor that mediates inflammatory responses. Previous studies have revealed that HO-1 inhibits NF-κB and attenuates inflammation (133), directly interferes with the nuclear localization signal of NF-κB (134), and inhibits activation of NF-κB by regulating the GSK-3β signaling pathway (135). HO-1 also inhibits the activation of NF-κB by inhibiting the expression of inflammatory factors, including TNF-α and IL-1β (136). Previous studies have further demonstrated that fenofibrate plays an important role in neuronal protection by increasing the expression of HO-1 and decreasing the expression of NF-κB after ICH (137).

**Figure 4:**
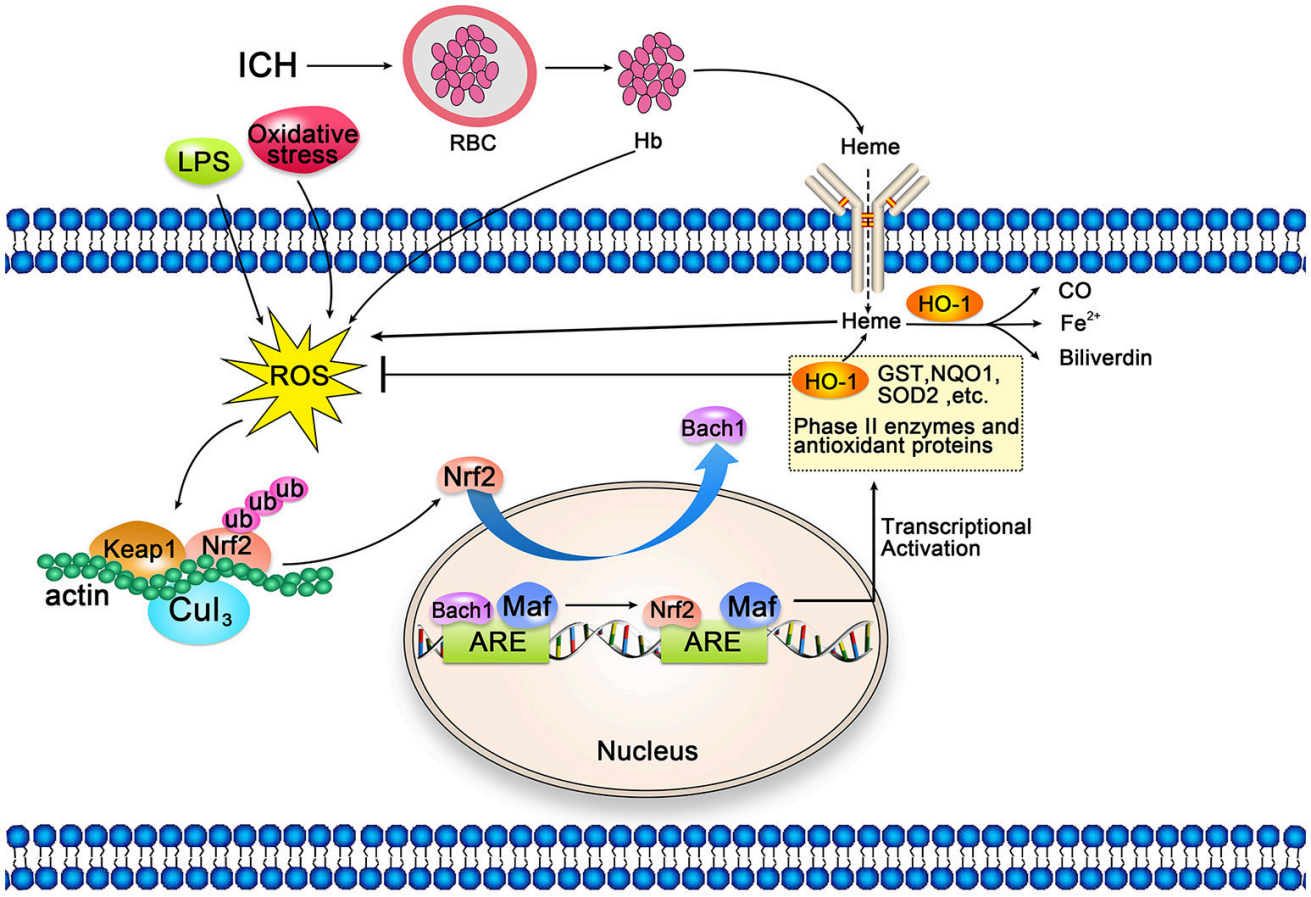
Schematic representation of cellular protection mechanism conferred by Nrf2-ARE pathway after intracerebral hemorrhage (ICH). Under normal conditions, Nrf2 forms a complex with actin-bound Keap1 and Cullin 3, which are isolated in the cytoplasm. Heme released after ICH, along with LPS and oxidative stress, can induce reactive oxygen species (ROS), which can induce and mediate dissociation of the Nrf2-Keap1 complex. Then, Nrf2 translocates to the nucleus and heterodimerizes with Maf. Subsequently, Nrf2 binds to antioxidant response elements (ARE) in the promoter regions of HO-1 and other target genes, activating transcription. Bach1 is an inhibitor of the Nrf2-ARE signaling pathway; thus, the removal of Bach1 is a prerequisite for transcription of this pathway.

Heme released by hematoma decomposition after ICH induces oxidative damage by producing ROS and reducing glutathione (GSH) antioxidant reserves (112). Nrf2 is a key modulator of the cellular defense mechanism against oxidative stress, which positively regulates the expression of HO-1 at the transcriptional level (138, 139). Under physiological conditions, Nrf2 is sequestered in the cytoplasm by binding to Kelch-like ECH-related protein 1 (Keap1) and Cullin 3 (Figure 4). Under oxidative stress, conformational changes in Keap1 lead to the release of Nrf2, which allows Nrf2 to be transferred to the nucleus. Nuclear-translocated Nrf2 binds to the antioxidant response element located in the promoter region of the cell protection gene, and activates the transcription of antioxidant genes, including *HMOX1* (138–140). This activation then leads to the production of phase II enzymes and antioxidant proteins, including HO-1, superoxide dismutase 2 (SOD2), chloramphenical acetyl transferase (CAT), glutathione S-transferase (GST), and NAD(P)H quinone dehydrogenase 1 (NQO-1) (141–143). During this process, Bach1 competes with Nrf2 for binding with Maf proteins (Bach1/Maf/ARE), resulting in decreased expression of antioxidant enzymes. Bach1 negatively regulates Nrf2-mediated transcription of antioxidant genes, and the removal of Bach1 is a prerequisite for transcriptional activation of antioxidant genes such as *HMOX1* (144, 145). Wang et al. (146) demonstrated that knockout of the *Nrf2* gene leads to decreased neurological function in mouse models if ICH, relative to levels observed in control mice. These findings indicate that Nrf2 may alleviate ICH-induced early brain damage by protecting against leukocyte-mediated free radical oxidative damage.

Yang et al. (147) demonstrated that hemoglobin induces upregulation and nuclear translocation of Nrf2 in astrocytes, which may contribute to upregulation of HO-1 and thereby increase the ability to defend against heme-induced toxicity. Since heme is a product of hemoglobin degradation, this phenomenon can be regarded as an adaptive self-defense mechanism in the pathological process of ICH. Therefore, the Nrf2-HO-1 pathway can be used as a target for neuroprotection after ICH. Chen-Roetling et al. first proposed the use of Nrf2 activators for the treatment of ICH (45). The main Nrf2 activators include sulforaphane (148, 149), curcumin (150, 151), hemin (23), dimethyl fumarate (DMF) (152, 153), and tert-butylhydroquinone (TBHQ) (154). The beneficial effects of these Nrf2 activators have been validated in rodent models of ICH induced via collagenase or autologous blood injection (45). DMF and hemin have long been approved for the treatment of psoriasis (45, 155) and acute porphyria (156, 157), respectively, which may provide insight into the clinical application of Nf2 activators for the treatment of ICH.

The results of the aforementioned studies indicate that HO-1 not only exerts anti-inflammatory and anti-oxidant effects via its catalytic products bilirubin and CO, but also plays an anti-inflammatory role by inhibiting the expression of inflammatory factors and blocking the NF-κB signaling pathway. Such studies have also demonstrated that Nrf2 regulates HO-1 expression by enhancing its anti-inflammatory and anti-oxidant effects. Thus, regulating the NF-κB and Nrf2-HO-1 signaling pathways may represent a novel strategy for ensuring neuroprotection following ICH.

### The relationship between HO-1 and ICH-related risk factors

Most cases of ICH are caused by hypertension, arteriosclerosis, vascular rupture, and obesity, the latter of which is the main risk factor for diabetes, cardio-cerebrovascular diseases, and hypertension. Several studies have indicated that the induction of HO-1 and its catalytic products reduces blood pressure, delays the development of hypertension (43, 158), and reduces damage to the target organ in models of cardiovascular and cerebrovascular disease. In addition, chronic induction of HO-1 reduces body weight and corrects hyperglycemia and hyperinsulinemia (158). Zhao et al. (159) demonstrated a unique role of HO-1 in anti-oxidative stress, anti-inflammation, anti-apoptosis, and enhancement of autophagy in mouse models of diabetes with high expression of HO-1, Tg-HO-1, and mutant HO-1 (Tg-mutHO-1).

Cerebral vasospasm is one of the main causes of death after ICH. Several previous studies have revealed that HO-1 exerts anti-vasculopathy effects (18, 160–162). Suzuki et al. (162) used mouse models to confirm that inducing gene expression of HO-1 exerts anti-vasospasm effects, which are important for the prevention and treatment of delayed cerebral vasospasm. Overexpression of HO-1 after experimental SAH can inhibit heme-induced arterial contraction and reduce vasospasm (18). Moreover, previous studies have indicated that introduction of the 11R-HO-1 protein into cerebral arteries prevents vasospasm, which may aid in the development of novel treatment strategies for ICH (160).

## Outlook

One major intriguing question regarding ICH treatment remains: Is there a time window during which ICH treatment must be applied to achieve significant results, similar to findings observed for ischemic stroke? Further, how should this time window be judged and defined? Because HO-1 expression is rapidly induced following ICH, exhibiting dynamic changes in structure and function throughout the course of the disease, it represents a highly promising entry point for determining whether such a time window exists. Indeed, HO-1 expression may be an appropriate biomarker for determining not only the time window for ICH treatment, but also patient prognosis. Elucidating the intrinsic mechanisms underlying the effects of HO-1 during ICH may enable the development of therapeutic strategies for ICH. Future studies should explore the following aspects: (a) the effect of HO-1 on microglial polarization after ICH; (b) HO-1 expression in different types of microglia and its influence on ICH prognosis (potentially via transcriptomics and proteomics); (c) gene-based interventions aimed at regulating HO-1 protein expression; (d) targeting transcriptional activators of HO-1, such as the Nrf2/Keap1 pathway, to affect HO-1 protein expression; (e) targeting transcriptional repressors of HO-1, such as Bach1 and micro-RNAs (miR217 and miR377), to affect HO-1 protein expression; (f) the multiple pathways that jointly regulate the level of HO-1 protein expression. At present, the key mechanism underlying secondary impairments in neurological function following ICH remain unknown, and there are no adequately effective treatments for ICH. Therefore, future studies should focus on multiple possible intervention targets. These in-depth studies of HO-1 will provide insight into novel strategies for the preservation of neurological function following ICH.

## Author contributions

Q-QL drafted the manuscript. JW repeatly modified the text structure and details. L-JL, X-YW, and Y-YS participate in the literature review and discussion about article writing and revision. All the authors revised and approved final version of the manuscript.

### Conflict of interest statement

The authors declare that the research was conducted in the absence of any commercial or financial relationships that could be construed as a potential conflict of interest.
